# Effect of monotherapy with darunavir/cobicistat on viral load and semen quality of HIV-1 patients

**DOI:** 10.1371/journal.pone.0196257

**Published:** 2018-04-24

**Authors:** Miguel A. López-Ruz, Miguel A. López-Zúñiga, María Carmen Gonzalvo, Antonio Sampedro, Juan Pasquau, Carmen Hidalgo, Javier Rosario, Jose Antonio Castilla

**Affiliations:** 1 Unidad de Enfermedades Infecciosas, Hospital Universitario Virgen de las Nieves, Granada, Spain; 2 Instituto de Investigación Biosanitaria de Granada (ibs.GRANADA), Granada, España; 3 UGC Medicina Interna, Complejo Hospitalario de Jaén, Jaén, Spain; 4 Unidad de Reproducción, UGC Laboratorio Clínico y UGC Obstetricia y Ginecología, Hospital Universitario Virgen de las Nieves, Granada, Spain; 5 UGC Microbiología, Hospital Universitario Virgen de las Nieves, Granada, Spain; 6 Dpto. Anatomía y Embriología Humana, Facultad Medicina, Universidad de Granada, Granada, Spain; Imperial College London, UNITED KINGDOM

## Abstract

Many patients previously using darunavir/ritonavir (DRV/r) (800/100mg) have switched to darunavir/cobicistat (DRV/C) (800/150 mg) either as part of triple therapy (ART) or as monotherapy with DRV (mDRV). The latter approach continues to be used in some countries for patients receiving long-term treatment. However, to date, the behaviour of DRV/C in the seminal compartment has not been analysed. This study explores how the combination behaves in monotherapy, with respect to the control of viral load and seminal quality. To this end, we studied 20 patients who were treated with mDRV/C after previous treatment with mDRV/r for at least 24 weeks. A viral load control in seminal plasma similar to that published in the literature was observed after 24 weeks of treatment with mDRV/C (viral load positivity in 20% of patients). Similarly, semen quality was confirmed (70% normozoospermic) in patients treated with this formulation, as has previously been reported for ART and mDRV/r. The DRV levels measured in seminal plasma were above EC_50_, regardless of whether the seminal viral load was positive or negative. We conclude that this mDRV/C co-formulation behaves like mDRV/r in seminal plasma in terms of viral load control and semen quality.

## Introduction

Sexual transmission is the main route of infection by human immunodeficiency virus type 1 (HIV-1). However, the risk of transmission via this route in patients who are positive for HIV-1, who are receiving antiretroviral treatment with ART and who achieve undetectable plasma viral load, provided there is correct adherence to the ART and the patient currently has no other sexually transmitted disease, is close to zero (1:100,000) [1]. Similarly, transmission between serodiscordant couples is significantly reduced when the seropositive member receives effective ART [2,3].

The semen quality of patients who are positive for HIV-1 and receiving treatment with ART is known to be impaired [4,5]. A similar decrease has been observed in HIV-1 patients who continue treatment with monotherapy with darunavir/ritonavir (mDRV/r) (800/100mg) [6].

The noninferiority of monotherapy with protease inhibitors enhanced with ritonavir to ART treatment has not been established, according to various meta-analyses [7,8] and systematic reviews [9]. Some of the studies included in these reviews were performed with DRV/r [10–14]. However, in real life the use of enhanced DRV in monotherapy has become widespread, due to its ease of application, the non-emergence of resistance, the maintenance of undetectable viral load in most patients and the economic savings achieved [15,16]. Nevertheless, only the Gesida [17] and EACS [18] guidelines admit their use in certain circumstances, while others, such as IAS [19] and DHHS [20], do not accept this approach.

Fewer studies have been conducted of mDRV/C (800/150 mg) [21,22]. The change from DRV/r to DRV/C was motivated by the findings of the study GS-US-216-230 [23] in which the efficacy and safety of the components were evaluated separately, mainly in naive patients, who were administered initial ART with DRV/C + 2 ITIAN. The results obtained were similar to those found in the ARTEMIS [24] and ODIN [25] studies.

Very few studies have examined the question of the seminal reservoir in patients receiving monotherapy with protease inhibitors combined with ritonavir [6,26] and our review of the literature did not reveal any that explored the effect of the DRV/C combination in monotherapy, with respect to viral load and to the semen quality of HIV-1 positive patients. To our knowledge, neither have any studies been conducted to evaluate DRV levels in semen with this combination.

The purpose of the present study is to evaluate semen quality, viral load and DRV levels in seminal plasma among patients who initiated treatment with mDRV/r, who have received at least six months’ treatment with mDRV/C and who present undetectable plasma viral load.

## Materials and methods

### Patients

Twenty HIV-1 patients aged over 18 years were drawn from those attending the outpatient infections clinic at Virgen de las Nieves University Hospital (Granada, Spain). These patients had received mDRV/C for 24 weeks and had previously been treated with mDRV/r for at least 24 weeks. All 20 patients had undetectable plasmatic viral load (<20 copies/mL) for a minimum of six months before starting mDRV/C. The following exclusion criteria were applied: the presence of sexually transmitted infection; any active disease at or during follow-up, including acute or chronic hepatitis B; history of virological failure to regimens based on protease inhibitors; history of illness that could provoke a decrease in semen quality, except for HIV infection itself; concomitant use of drugs with potential interaction with the prescribed drugs; low adherence to the prescribed therapy.

**Fig 1:**
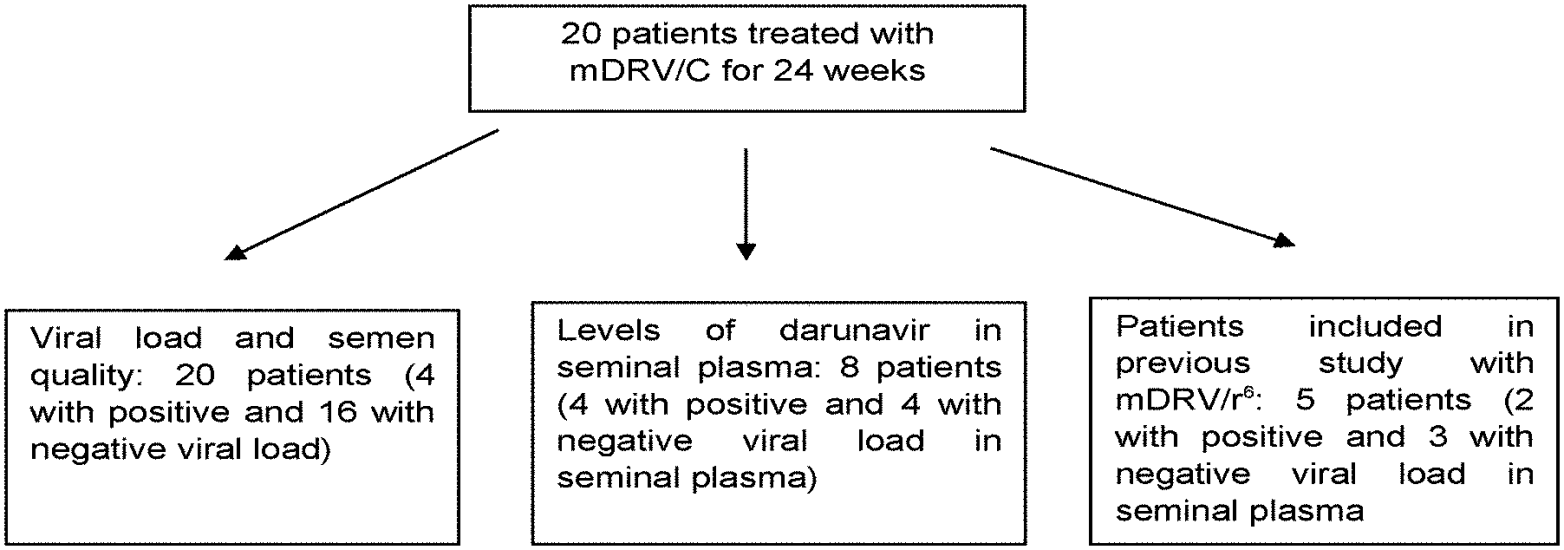
Patients included in different procedures of the study according to the seminal viral load.

From these 20 patients, eight were selected, four with positive viral load in semen and four with negative viral load, to determine the concentration of DRV in seminal plasma (Fig 1). All these patients provided signed informed consent to participate in the study, in accordance with the principles of the Declaration of Helsinki. In addition, the study was approved by the research ethics committee of Granada (CEI Granada). The patients’ data were codified to maintain anonymity, as required by Spanish data protection laws.

The results for viral load and semen quality of these 20 patients, after treatment with mDRV/C for 24 weeks, were compared with those obtained in a previous study performed in 21 patients who after receiving at least 24 weeks of ART, were given mDRV/r for 48 weeks [6]. Of the 20 patients with mDRV/C, five had previously participated in this previous study of mDRV/r [6] (Fig 1).

The patients received the normal medical visits, every four months. Adherence to the medication was monitored by pharmacy report and by asking the patient during the medical visit.

### Variables analysed

Age, androgenic history, reproductive history, CD4+, CD4+ nadir, HIV viral load in seminal fluid and plasma. The following semen quality parameters were analysed: viscosity, appearance, liquefaction, volume, pH, sperm concentration, percentages of progressive and total motility and of live sperm.

### Semen analysis

All the samples were collected by masturbation, after 3–4 days of sexual abstinence, in the andrology laboratory of the Human Reproduction Unit at the Virgen de las Nieves University Hospital. On delivery of the sample to the laboratory, the following data were recorded: period of sexual abstinence, whether any loss of the ejaculate had occurred, medication taken by the participant, recent febrile processes, body mass index and recent extreme sports activity. All the samples were analysed within one hour of collection, by a single highly-trained observer, with more than ten years’ professional experience, who regularly participated in internal and external controls. In this way, between-observer variation was avoided and the accuracy of the study was ensured.

The ejaculates were analysed according to WHO recommendations [27] and the SEMQUA 2014 [28] international guidelines for semen quality studies. The concentrations of sperm and of round cells were determined using a Neubauer Improved haemocytometer and positive displacement pipettes. The total number of sperm cells was estimated by multiplying the concentration by the seminal volume. The seminal volume was measured by weighing the sample. Sperm motility was evaluated visually to calculate the percentage of sperm with progressive motility. The vitality, the percentage of normal forms and the teratozoospermia index were also calculated. The percentage of participants whose results were below the lower limit of the WHO 2010 reference values for sperm parameters was determined.

### Determination of viral load in seminal fluid

The viral load in seminal fluid was determined after centrifugation at 3000g for 15 min. The technique used to quantify HIV-1 RNA in the final processed sample was that of real-time PCR, an adaptation of the HIV-1 COBAS Ampliprep/Taqman assay version 2.0 (Roche).

### Determination of DRV levels in seminal fluid

DRV, C_trough_ and seminal plasma concentrations were quantified using a modified and validated liquid chromatography with a tandem mass spectrometry method [29]. The participants were instructed to take their drugs in the morning after a standard breakfast and to record the time when they had taken the medication the previous day. The semen samples were collected 24 ±1 h after the previous mDRV/C dose. Seminal plasma was separated by centrifugation and stored at -80°C until tested. The standard curves were highly linear over the range of 10−1000 ng/mL in seminal plasma. The intra- and interassay coefficient of variation were <15% for DRV in seminal plasma.

### Statistical analysis

A descriptive analysis was performed to obtain measures of central tendency and dispersion for the numerical variables, and absolute and relative frequencies for the categorical ones. The Shapiro-Wilks test of normality was applied. Differences between the groups were determined by a bivariate analysis, using the non-parametric Mann-Whitney test for the numerical variables and Pearson’s chi-square test or Fisher’s exact test for the categorical ones. The level of significance considered for all analyses was p<0.05. The statistical analysis was performed using IBM SPSS Statistics 19 software.

## Results

### General characteristics of the patients included in the study

The mean age was 47.5 years (SD: 6.8), the mean CD4^+^ T cell count was 882 cell/μL (SD:316.8) and the mean nadir CD4^+^ T cell count was 277 cell/μL (SD:191.5).

### Viral load in seminal fluid

HIV-PCR in the seminal fluid was analysed for all 20 patients; four (20%) were positive (>20 copies/mL), with a range of 21–1070 copies/mL.

### Semen quality

The data on semen quality for patients on monotherapy are summarised in Tables 1 and 2. Only 14 of the 20 samples (70%) patients presented higher sperm values than the lower limit of the WHO 2010 reference values. The alterations most frequently observed were oligoastenospermia and oligoastenoteratospermia (Table 2). No significant differences were observed in the sperm parameters analysed in patients treated with ART and mDRV/r [6] with respect to the values obtained in the present study with mDRV/C (Table 2).

**Table 1:** Seminal fluid values of the 20 patients treated with mDRV/C compared with the results of 21 patients treated initially with ART and then with mDRV/r [6].

	ART n = 21 [6]	mDRV/r n = 21 [6]	mDRV/C n = 20
Volume; Ml	2.3(1.7–3)	1.9(1.5–3.4)	2.5(1.5–3.5)
PH	7.5(7.2–7.5)	7.5(7.2–7.7)	7.6(7.5–7.7)
Sperm; x106/mL	59(20.5–141.5)	39(14–109.5)	43(13.3–99)
Round cells; x106/mL	3(1–8)	2(1.1–3.8)	2(1–6)
Total sperm count; x106	154(42–319.5)	132(31.8–259.5)	122.8(26.4–251.2)
Progressive motility; %	45(32.5–63)	52.5(40–65)	50(41–64)
Total motility; %	60(42–70)	60(45–79)	64(60–81)
Normal form; %	5(3–6)	4(4–7.5)	6(5–9)
Live sperm;%	88(79.5–90.5)	86.5(80.5–90.8)	85(79–89)
Teratospermia index	1.3(1.2–1.4)	1.3(1.2–1.4)	1.3(1.2–1.5)

Median (Q1-Q3); ART: Triple therapy; mDRV/r: monotherapy with darunavir/ritonavir; mDRV/C: monotherapy with darunavir/cobicistat; Not significant

**Table 2:** Semen quality in patients receiving mDRV/r [6] and mDRV/C.

Semen quality	mDRV/r n = 21	mDRV/C n = 20
Normal	13(61.9%)	14(70%)
Oligoteratospermia	2(7.1%)	0(0%)
Oligoastenospermia	2(7.1%)	2(10%)
Oligoastenoteratospermia	3(10.8%)	3(15%)
Teratozoospermia	4(14.3%)	0(0%)
Astenozoospermia	0(0%)	0(0%)
Oligozoospermia	1(3.5%)	1(5%)

mDRV/r: monotherapy with darunavir/ritonavir; mDRV/C: monotherapy with darunavir/cobicistat; Not significant

Of the patients included in both studies (n = 5) three had no positive viral load in seminal plasma with no monotherapy regimen, one had viral load in both regimens and one had positive viral load with mDRV/C and negative viral load with mDRV/r (data not shown).

Comparison of viral load positivity of the seminal plasma among patients with mDRV/C (20%) with respect to those with mDRV/r (3%) revealed no significant differences (p = 0.0656). Neither were any differences observed regarding the average number of copies (382/mL vs. 132/mL, p = 0.480).

Semen quality was normal in 16/28 (57.1%) of patients in ART, in 13/21 (61.9%) of those in mDRV/r and in 14/20 (70%) of those in mDRV/C. No significant differences were observed in comparisons of ART with mDRV/r (p = 0.7372), ART with mDRV/C (p = 0.3643) or mDRV/r with mDRV/C (p = 0.5848).

The median concentration of DRV in seminal fluid was 489.4 ng/mL (range: 204.4–1889.2 ng/mL), with 680.5 ng/mL in the four patients with positive viral load and 405.9 ng/mL in the four with negative viral load (p = 0.149) (data from individual patients not shown).

## Discussion

To our knowledge, this study is the first to analyse semen quality and viral load in HIV+ men treated with mDRV/C. To date, all seminal plasma studies of monotherapy in HIV+ patients have been performed using ritonavir, at low doses, as a pharmacokinetic enhancer. The mDRV/C treatment combination was adopted because of the potency of the DRV/C [23] combination, and experience in the use of monotherapy in conjunction with protease inhibitors [15].

In our study, 20% of the patients treated with mDRV/C had a negative plasma viral load and a positive seminal viral load, a similar proportion to that observed in other studies of patients treated with ART [26,30–32] or protease inhibitors/r [33]. However, our findings differ from those obtained by some authors [34,35], who did not detect any patient with a positive seminal viral load. These discrepancies may be due to the HIV-RNA detection technique used, since the detection limits described by the latter authors were much higher (<150 copies/mL and <200 copies/mL, respectively) than those used in our study (<20 copies/mL).

Furthermore, the viral load of RNA-HIV found in the seminal plasma of patients treated with mDRV/C presented values similar to those observed by other authors with 270–1345 copies/Ml [33], with 31–1445 copies/Ml [26] or with 35–1210 copies/Ml. [6] One patient in the current study presented a positive viral load in seminal plasma after 24 weeks with mDRV/C. This patient had previously presented a positive viral load when treated with mDRV/r for 48 weeks.

The levels of DRV observed in seminal fluid are similar to those found by other authors in patients with similar treatments [33,36]. The latter studies reported that the free fraction of DRV in semen ranged from 7–20% of that observed in blood plasma. The free fraction of the drug is considered the only effective fraction available for tissue diffusion or penetration [33]. In view of the low percentage found of positive viral load with mDRV/C, we believe that this drug is as safe and effective as ART, despite the low penetration of DRV within the male genital tract. The capacity of a drug to enter the male genital compartment depends on a number of factors, including molecular size, lipophilicity, degree of ionisation, plasma protein binding and whether or not the drug is a substrate for efflux transporters [37]. In general, PIs present various unfavourable characteristics, which tend to impede passive diffusion [36,38]. Like most PIs, DRV is highly bound to plasma proteins (95%), with a small fraction (5%) of unbound drug available to penetrate the male genital tract.

The levels of DRV in the seminal plasma of the eight patients in our study were above EC_50_ [36] and EC_90_ [39], independently of whether the viral load in semen was positive or negative. Although we are unaware of previous studies of seminal plasma concentrations with mDRV/C, with which to compare our results, it has been reported that Cmin in blood plasma is adequate even in the presence of a positive viral load in seminal plasma [40]. Other authors have also observed an absence of relationship between ART levels and viral load [41].

The standardisation of seminal quality studies is of crucial importance if valid conclusions are to be obtained. Our group participated in the development of the SEMQUA recommendations for the design and evaluation of seminal quality studies [28] and the present analysis is in line with these recommendations.

Regarding seminal quality, no significant improvement was observed with mDRV/C with respect to mDRV/r or ART, with normozoospermic patients accounting for 70%, 62% and 57%, respectively, of the total. However, the observed pattern of improving seminal quality in patients who received mDRV/r and mDRV/C may be due to the fact that the nucleoside analogues received by patients in antiretroviral treatment are associated with greater mitochondrial toxicity [42,43].

The main limitation of this study is the limited number of cases examined, regarding both viral load and seminal quality, and the number of determinations of DRV concentration in seminal plasma. Accordingly, our findings are subject to confirmation by subsequent studies with a larger number of participants. A second limitation is the centrifugation step performed to separate the seminal plasma samples. Unrecognized cell contamination of the seminal plasma samples could confound our results and might result in inhibition of PCR and false HIV-RNA measurements, despite the centrifugation step. We believe this scenario is unlikely, however, as we observed no inhibition of PCR in the 20 samples analysed. Furthermore, the proportion of HIV-1 infected men with sustained suppressed HIV-1 RNA in blood plasma who have detectable levels of HIV-1 RNA in seminal plasma, as commented above, was similar to that observed by other authors using a different centrifugation protocol [26,30–33].

Despite its limitations, the present study provides suggestive evidence that in HIV-1 patients treated with mDRV/C, viral load and seminal quality results are similar to those obtained in the previous study in this respect [6], both in the treatment with ART and in that with mDRV/r. These results need to be confirmed by a study with a larger number of patients.
